# Highly effective, regiospecific reduction of chalcone by cyanobacteria leads to the formation of dihydrochalcone: two steps towards natural sweetness

**DOI:** 10.1186/s12934-017-0752-3

**Published:** 2017-08-04

**Authors:** Beata Żyszka, Mirosław Anioł, Jacek Lipok

**Affiliations:** 1 Department of Analytical and Ecological Chemistry, Faculty of Chemistry, University of Opole, Oleska 48, 45-052 Opole, Poland; 2 Department of Chemistry, Faculty of Food Science, Wroclaw University of Environmental and Life Sciences, Norwida 25, 50-375 Wroclaw, Poland

**Keywords:** Chalcone, Biotransformation, Cyanobacteria, Dihydrochalcone, Regioselective bio-reduction

## Abstract

**Background:**

Chalcones are the biogenetic precursors of all known flavonoids, which play an essential role in various metabolic processes in photosynthesizing organisms. The use of whole cyanobacteria cells in a two-step, light-catalysed regioselective bio-reduction of chalcone, leading to the formation of the corresponding dihydrochalcone, is reported. The prokaryotic microalgae cyanobacteria are known to produce phenolic compounds, including flavonoids, as natural components of cells. It seems logical that organisms producing such compounds possess a suitable “enzymatic apparatus” to carry out their biotransformation. Therefore, determination of the ability of whole cells of selected cyanobacteria to carry out biocatalytic transformations of chalcone, the biogenetic precursor of all known flavonoids, was the aim of our study.

**Results:**

Chalcone was found to be converted to dihydrochalcone by all examined cyanobacterial strains; however, the effectiveness of this process depends on the strain with biotransformation yields ranging from 3% to >99%. The most effective biocatalysts are *Anabaena laxa*, *Aphanizomenon klebahnii*, *Nodularia moravica*, *Synechocystis aquatilis* (>99% yield) and *Merismopedia glauca* (92% yield). The strains *Anabaena* sp. and *Chroococcus minutus* transformed chalcone in more than one way, forming a few products; however, dihydrochalcone was the dominant product. The course of biotransformation shed light on the pathway of chalcone conversion, indicating that the process proceeds through the intermediate *cis*-chalcone. The scaled-up process, conducted on a preparative scale and by using a mini-pilot photobioreactor, fully confirmed the high effectiveness of this bioconversion. Moreover, in the case of the mini-pilot photobioreactor batch cultures, the optimization of culturing conditions allowed the shortening of the process conducted by *A. klebahnii* by 50% (from 8 to 4 days), maintaining its >99% yield.

**Conclusions:**

This is the first report related to the use of whole cells of halophilic and freshwater cyanobacteria strains in a two-step, light-catalysed regioselective bio-reduction of chalcone, leading to the formation of the corresponding dihydrochalcone. The total bioconversion of chalcone in analytical, preparative, and mini-pilot scales of this process creates the possibility of its use in the food industry for the production of natural sweeteners.

## Background

Chalcones (1,3-diphenyl-2-propen-1-ones) are the biogenetic precursors of all known flavonoids (derivatives of 2-phenyl-benzo-gamma-pyrone), which play an essential role in various metabolic processes in photosynthesizing organisms [1, 2]. As phytochemicals of a lipophilic nature, chalcones are composed of two aromatic rings linked by a three-carbon α,β-unsaturated carbonyl system, which usually results in their yellow colour. Chalcones considered as obligate intermediated in flavonoid biosynthesis but they do not accumulate to appreciable degree in most plants [3]. They are especially abundant in fruits (e.g., citruses, apples), vegetables (e.g., tomatoes, shallots, bean sprouts, potatoes), spices (e.g., licorice), and are also widely distributed in various parts (roots, rhizomes, heartwood, buds, leaves, flowers, and seeds) of species of genera *Angelica*, *Sophora*, *Glycyrrhiza*, *Humulus*, *Scutellaria*, *Parartocarpus*, *Ficus*, *Dorstenia*, *Morus*, *Artocarpus*, and so forth—many of which have been used for centuries in traditional herbal medicine [4, 5]. Both natural and synthetic compounds with a chalcone-based structure have received great attention due to their wide variety of pharmacological activities [6, 7]. Because of the health promoting properties, an increasing interest in finding new biologically active derivatives of chalcone has been recently observed [8–10].

Extraction from plants is classic and still the most popular method of obtaining the majority of flavonoids, including chalcones. However, this method suffers from the low concentration of these compounds in plant tissues, as well as from environmental, seasonal, or regional variations of their abundance in these natural resources [11]. The second extraction method, chemical synthesis, requires extreme reaction conditions and toxic chemicals, which constrains its application on an industrial scale [12]. Biocatalysis seems to offer an interesting alternative to both the aforementioned approaches, especially when it involves whole microbial cell systems that offer scalable, cheaper and more accessible strategies for efficient acquisition of these compounds [13]. Simultaneously, the use of biocatalysis minimizes the problems of isomerization, racemization, epimerization and rearrangement during the transformation [14, 15].

Studies on the interactions of microorganisms with chalcones published thus far show that the transformation of these compounds can only be done by heterotrophic microorganisms, such as bacteria, filamentous fungi and yeast. Biotransformations of chalcones by whole cells of these microorganisms are similar to the metabolic conversion pathways of these compounds in plants and include hydroxylation [16–21], cyclization [16, 17, 19–22], O-demethylation [16, 17, 19], O-dealkylation [18], epoxidation [22], glycosylation [23–25], and hydrogenation [19, 20, 26–30]. The reduction of a dihydrochalcone to the respective alcohol has been rarely reported [27–30]. Moreover, microorganisms are able to cleave the *C*-ring of the benzo-γ-pyrone system of flavonoids to form chalcones [31, 32]. It should be noted that there is no information about the natural occurrence of flavonoids in the cells of heterotrophs that can transform these substances. Interestingly, however, these microorganisms contain an assembly of biosynthetic genes and are able to form an artificial phenylpropanoid pathway, which may allow them to form flavonoids and related compounds [33, 34].

The microbial transformation of compounds containing an α,β-unsaturated chain connected to a carbonyl group has gained growing interest, especially with the recent rediscovery of the Old Yellow Enzyme (OYE) [35, 36] and its homologues from yeast, bacteria, and plants [37, 38]. Enoate reductases catalyse a highly stereoselective reduction of a broad variety of α,β-unsaturated compounds, producing excellent yields [35, 39, 40]. A recombinant enoate reductase was also expressed in a prokaryotic microalgae, cyanobacteria, and was used for the light-induced, enantioselective reduction of C=C bonds [41]. Such a transformation leads to the formation of dihydrochalcones. Due to their sweet taste and natural origin, these compounds are desirable in the food industry for the production of multicomponent, low-calorie sweeteners. In turn, their antioxidant, UV-protective and pro-health properties make dihydrochalcones interesting to the pharmaceutical and cosmetics industries [42, 43].

Little information is available regarding the connections between flavonoids and cyanobacteria. Known examples are related to the presence of flavonoids in the cells and culture media of cyanobacteria [44–46]; cyanobacterial cells growing in different culture media can accumulate flavonoids to near-millimolar levels [47]. Additionally, evidence was found for the influence of flavonoids on gene expression of cyanobacteria [48, 49] and the ability to inhibit the growth of cyanobacteria by plants containing flavonoids [50–52]. Nevertheless, the interactions between chalcones, the initial precursors of flavonoid biosynthesis, and cyanobacteria still remain poorly understood, especially due to unknown mechanisms(s) of metabolic adaptations of these microorganisms. Therefore, the study of such interactions seems reasonable.

Due to the potential ability of cyanobacteria to promote biotransformation of flavonoids, we decided to study such processes using chalcone as the model compound. Whole cells of halophilic and freshwater cyanobacteria strains were used to indicate the favoured routes of chalcone biocatalytic transformation in batch cultures performed on analytical, preparative and mini-pilot scales.

## Methods

### Chemicals

Chalcone (Cat. No. 136123) was purchased from Sigma-Aldrich (Poznan, Poland). The chalcone stock solution was prepared in dimethyl sulfoxide (DMSO) (15 mg/mL) and was sterilized by filtration, prior to its addition to the medium. The chalcone stock solution was prepared on the same day of its use and was stored in the dark to prevent photodamage. All chemical components of the medium were purchased from POCH S.A. (Avantor Performance Materials Poland S.A., Gliwice, Poland). All chemicals were of analytical grade. Milli-Q water (Merck, Millipore, Germany) was used for culture preparation and maintenance, whereas special Millipore Milli-Q A10 water of high purity was used for any analytical purposes.

### Strains and culture conditions

Cyanobacteria used in our experiments occur either naturally in hypersaline ponds, such as *Spirulina platensis* [strain C1 (PCC9438)], or in freshwater ecosystems, such as *Anabaena* sp. [strain CCALA 007], *Anabaena laxa* [strain CCALA 805], *Aphanizomenon klebahnii* [CCALA 009], *Nodularia moravica* [strain CCALA 797], *Chroococcus minutus* [strain CCALA 055], *Merismopedia glauca* [strain CCALA 099] and *Synechocystis aquatilis* [strain CCALA 190]. Axenic culture of the *S. platensis* strain was obtained from the Pasteur Culture Collection (PCC) (Institut Pasteur, Paris), whereas all other cyanobacterial species were purchased from the Culture Collection of Autotrophic Organisms (CCALA) (Institute of Botany AS CR, Trebon, Czech Republic).

To make the inoculates, which were used to initiate the experimental cultures, all tested microorganisms were pre-grown in standard media: MSp (ATCC 1679) medium for the halophilic strain, and BG11 (ATCC 616) or Z8 medium for freshwater strains.

Subcultures of cyanobacteria were revitalized every 3 weeks by transferring 10-mL aliquots to 50 mL of fresh adequate media [53]. The cultures of the tested cyanobacteria were grown at 24 ± 1 °C for 16 h day (1000 lx light intensity) and 8 h night photoperiods, corresponding to the conditions of the long day in 250 mL Erlenmeyer flasks containing 60 mL of each culture [54, 55].

### Experimental cultures

Screening (analytical) scale biotransformations of chalcone were arranged by transferring the appropriate volumes of aliquots from 21-day-old subcultures to adequate, fresh media. The volumes of the inoculates were established experimentally by considering the final concentration of chlorophyll, which was 1 mg/L at the beginning of culturing for all tested species. Chlorophyll content was measured in methanolic extracts, according to a previously described procedure [56]. The inoculum was added to 100 mL Erlenmeyer flasks containing 30 mL of the appropriate culture medium supplemented with chalcone stock solution (0,13%, v/v), which resulted in a final chalcone concentration of 20 mg/L. This concentration of substrate was chosen based on the results of screening experiments that were performed in a concentration range from 5 up to 100 mg/L, which guaranteed the highest amount of chemical that did not induce the sudden death of cyanobacterial cells.

Cyanobacterial cultures were incubated at 24 ± 1 °C under constant adjusted light conditions with a photoperiod (16 h:8 h, day:night at 2500 lx light intensity) of 14 days. The stability of substrate under these conditions was demonstrated by preparing and incubating control samples consisting of the solution of tested flavonoid in sterile cultivation medium, the substrate control. The culture controls consisted of the microbial colonies (inocula) in fermentation medium cultivated without the chalcone. All experiments, including controls, were performed at least in triplicate.

After fourteen (14) days of incubation, the cells and culture medium were separated by filtration followed by centrifugation at 5.000×*g* for 1 min. Next, all the transformation medium obtained in each repetition was removed separately and was extracted three times with 10 mL of ethyl acetate. Then, these three extracts (from each separate repetition) were combined and dried over anhydrous magnesium sulphate, and the solvent was evaporated to dryness using a vacuum evaporator. The remaining residue was dissolved in 200 µL of ethyl acetate and subjected to analysis with the use of planar chromatography (TLC) and gas chromatography (GC–MS), using the appropriate standards. The effectiveness of the biotransformations was calculated based on the quantification of the products obtained with respect to the areas of relevant peaks, while the total peak area was treated as 100%. The resulting biotransformation yield is the average of the values obtained.

Additionally, in the case of *A. klebahnii*, the entire transformation mixture was removed every 24 h, prepared as described above and analysed by GC–MS. The determination of the growth of *A. klebahnii* in experimental cultures was performed by time-course measurements of total chlorophyll content [53].

### Preparative-scale biotransformations of chalcone by cyanobacteria

To scale-up the biotransformation process, eight repetitions of the same experiment were performed in 1000 mL Erlenmeyer flasks containing 250 mL of the cultivation medium. The cultures were incubated under the same conditions using the same final concentration of substrate. After 14 days of incubation, the mixtures were extracted with ethyl acetate (each repetition, 3 × 80 mL) and dried over anhydrous MgSO_4_. After evaporation of the solvent in a vacuum evaporator, the products of transformation were separated by preparative thin layer chromatography (PTLC), and the structures of the separated substances were confirmed by mass spectrometry (MS), infrared spectroscopy (IR), proton nuclear magnetic resonance (^1^H NMR), and carbon nuclear magnetic resonance (^13^C NMR), including homonuclear correlation spectroscopy (COSY) and heteronuclear single-quantum correlation (HSQC) experiments.

### Biotransformation of chalcone by cyanobacterium *Aphanizomenon klebahnii* in a mini-pilot scale photobioreactor

The mini-pilot scale photobioreactor was composed of a “Jupiter,” modular, autoclavable fermentation unit and multicolour light emitting diodes (LED) module called “Elara.” Both modules are produced by Solaris (Mantova, Italy). “Jupiter” is designated for microbial and cell culture applications and is suitable for both aerobic and anaerobic bioprocesses. The total volume of the borosilicate glass chamber is 4 L; however, the working volume was set at 2 L. The chamber was equipped with a Teflon^®^ stirrer and cooling system. “Elara” contains sets of warm-white, red, green and blue LED lamps placed in the collar surrounding the glass chamber and padded with a reflective surface to lower the loss of light irradiation and to prevent the penetration of outside light. The entire system is controlled by the dedicated Process Control System based on Leonardo 2.0 software. Such a photobioreactor allows for the control of carbon dioxide supply, water supply, optimal temperature, efficient exposure to light, culture density, pH level, gas supply rate and the stirring regime of the culture.

The 2 L volume mini-pilot scale of *A. klebahnii* culture was established using pre-cultured inoculum and freshly autoclaved Z8 medium. The volume of inoculum was adjusted experimentally with respect to the concentration of chlorophyll, which at the beginning of culturing was 1 mg/L. The culture was supplemented with chalcone up to a final concentration of 20 mg/L and was irradiated with white/yellow light [(RGB)—R: 225, G: 242, B:87, S:255] of 2,5 ± 0,3 klux intensity with a defined photoperiod (16 h:8 h, day:night). The temperature of the culture was set at 25 °C, and the stirring speed was 100 rpm. Circulating, filtered air was supplied through a syringe filter to the bottom part of the vessel for uniform mixing of the culture and to prevent stagnation. This setup was left undisturbed for 7 days, the entirety of the batch experiment. A photobioreactor was connected to the additional unit, a gas analyser (produced by Solaris), which allowed for the measurement of oxygen and carbon dioxide concentrations in the headspace of the culture in the real-time mode. Those data were recorded, and the respiration coefficient was calculated.

The same parameters were applied to the sterile Z8 medium containing chalcone at a concentration of 20 mg/L but without cyanobacterial cells (without inoculum), which acted as a substrate control. The same parameters were also applied to the sterile Z8 medium inoculated with the biota under examination but without the chalcone that was treated as the culture control.

### Determination of biotransformation course and obtained products by chromatographic and spectroscopic methods

The biotransformations were carried out by TLC and GC. In the case of thin layer chromatography, the 10 µL volumes of ethyl acetate extracts from experimental cultures, substrate and culture controls, and the solution of chalcone standard were applied on TLC aluminium plates (silica gel 60, 0.2 mm thickness; Merck, Germany) using a CAMAG Linomat 5 applicator (CAMAG, Muttenz, Switzerland). Plates were developed in a dedicated glass chamber previously saturated with a hexane:acetone mobile phase mixture (3:1, v/v). Developed plates were visualised under 254 and 366 nm wavelengths of UV-light and under white light in a CAMAG TLC Visualizer 2 (CAMAG, Muttenz, Switzerland). Then, the plates were sprayed with a solution of 10 g of Ce(SO_4_)_2_ and 20 g of phosphomolybdic acid (H_3_[P(Mo_3_O_10_)_4_]) dissolved in 1 L of 10% H_2_SO_4_ to verify the presence of flavonoids, which give yellow to brown-orange spots. After spraying, the plates were gently heated until coloured spots appeared, whereupon the plates were inspected again under the UV light at 254 and 366 nm wavelengths and under white light in the reflectance mode. The retention coefficients (R_f_) and colours of the spots were recorded using CAMAG VisionCats software.

The changes in chemical composition of biotransformation medium were determined by the GC–MS system Thermo Scientific TriPlus Trace GC Ultra fitted with the ITQ 1100 mass detector (Thermo Fischer Scientific, Waltham, Massachusetts, USA) equipped with an HP-5 fused silica capillary column (30.0 m × 0.25 mm i.d., film thickness 0.25 µm). To determine the composition of the products the following temperature program was used: 90 °C/0 min, gradient 3 °C/min to 200 °C/5 min, gradient 10 °C/min to 280 °C/5 min. The injector temperature was set at 250 °C. The flow rate of the carrier gas (helium) was set at 1.5 mL/min. The split flow was 30 mL/min. A split ratio of 20:1 was applied. The chromatographic data were recorded and processed using the XCalibur software.

The products of biotransformations performed in preparative and mini-pilot scale experiments were separated by PTLC by the use of a CAMAG system and silica gel glass plates without a fluorescent indicator (Silica gel 60 matrix, 500 µm thickness, Sigma-Aldrich) using a hexane:acetone (3:1, v/v) mixture as the eluent. Separated products were extracted from the silica bed with ethyl acetate, which was then evaporated under a stream of nitrogen. Then, the structures of isolated products were confirmed by MS, FTIR and nuclear magnetic resonance techniques: ^1^H NMR, ^13^C NMR, HSQC and COSY. Spectra were recorded using the NMR Bruker UltraShield 400 MHz spectrometer with tetramethylsilane (TMS) as an internal reference. The NMR spectra were measured in DMSO-d6. To confirm the presence of characteristic absorption bands, the obtained products were incorporated in KBr pellets and were subjected to FT-IR measurements using a Thermo Scientific NICOLET 6700 spectrometer (Thermo Fischer Scientific, Waltham, Massachusetts, USA). The structures of the known compounds were confirmed by comparing their spectroscopic properties with spectra of appropriate standards and with data published in the literature.

## Results and discussion

### Bioconversion of chalcone by cyanobacteria in analytical scale

To select the strains capable of bio-converting chalcone, eight species of cyanobacteria, one halophilic strain (*S. platensis*) and seven freshwater strains (*A. laxa*, *Anabaena* sp., *A. klebahnii*, *N. moravica*, *C. minutus*, *M. glauca*, and *S. aquatilis*), were screened. These species are known to be capable of transforming monoterpenes and organophosphonic compounds [57]. To the best of our knowledge there are no reports regarding biotransformation of chalcones with the help of cyanobacteria. In fact, known reports describing hydrogenation of chalcones by microorganisms to the corresponding dihydrochalcones concerned the reactions catalyzed by bacteria of the genera *Gordonia*, *Lactobacillus*, *Rhodococcus*, yeasts of the genera *Rhodotorula*, *Saccharomyces*, *and* Yarrowia, and mycellial fungi of the genera *Aspergillus*, *Chaetomium*, *Fusarium* [19, 27, 28, 30].

All cyanobacterial strains examined were capable of transforming chalcone, and a few strains converted this substrate with a >99% yield upon fourteen (14) days of incubation. The mass of the molecular ion of the conversion product, its m/z fragmentation and the specific differences in its mass spectrum compared to chalcone, strongly suggested the propenyl chain was bio-reduced, which thus suggests dihydrochalcone was formed. The use of the GC–MS system with respect to the retention time and the specific fragmentation of dihydrochalcone used as a standard, as well as the comparison of spectroscopic data of this substance with those published in the literature, confirmed this hypothesis. Moreover, the results of the NMR experiments (^1^H, ^13^C, HSQC and COSY) confirmed the structure of dihydrochalcone as the product and led us to the same conclusion.

**Fig. 1:**
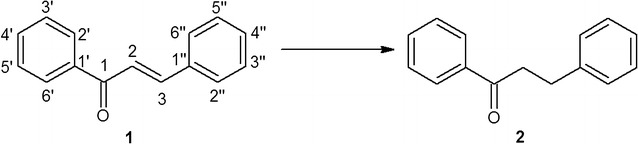
Regioselective bioreduction of chalcone (**1**) that leads to the formation of dihydrochalcone (**2**) as the only product, as it is catalysed by *Anabaena laxa*, *Aphanizomenon klebahnii*, *Nodularia moravica*, *Synechocystis aquatilis* and *Merismopedia glauca*

Based on this finding, supported by the GC–MS quantification, chalcone was found to be converted to dihydrochalcone by all examined cyanobacterial strains; however, the effectiveness of this process depends on the strain with biotransformation yields ranging from 3% to >99% (after 14 days of incubation). The most effective biocatalysts, *A. laxa*, *A. klebahnii*, *N. moravica*, *S. aquatilis* and *M. glauca*, reduced chalcone, causing alteration only in the C=C bond of the olefin fragment of the molecule without affecting the carbonyl group (Fig. 1).

The first four freshwater cyanobacterial strains completely transformed the substrate with >99% yield, whereas the latter strain transformed the substrate with 92% yield. It is worth noting that in all five strains, the regioselective reduction of 1,3-diphenyl-2-propen-1-one was the only means identified by which this substrate was transformed.

**Fig. 2:**
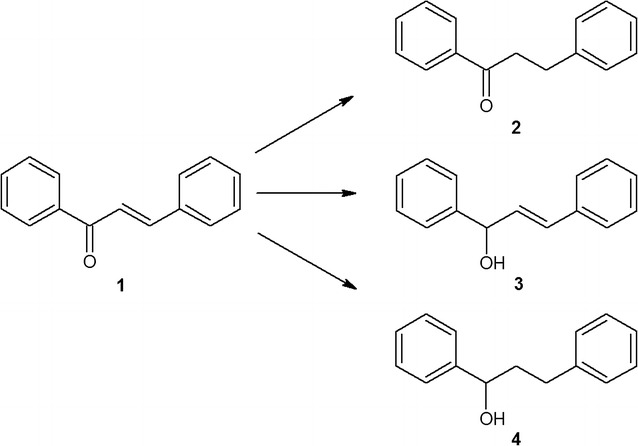
Specific transformation of chalcone catalysed by *Anabaena* sp.

Alternatively, the *Anabaena* sp. and *C. minutus* could transform chalcone using several means and thus provided few products. However, dihydrochalcone remained dominant. *Anabaena* sp. converted chalcone into dihydrochalcone with a yield of nearly 90%, producing additional corresponding secondary alcohols: 1,3-diphenyl-2-propen-1-ol and 1,3-diphenylpropan-1-ol with 7 and 6% of yields, respectively (Fig. 2).

**Fig. 3:**
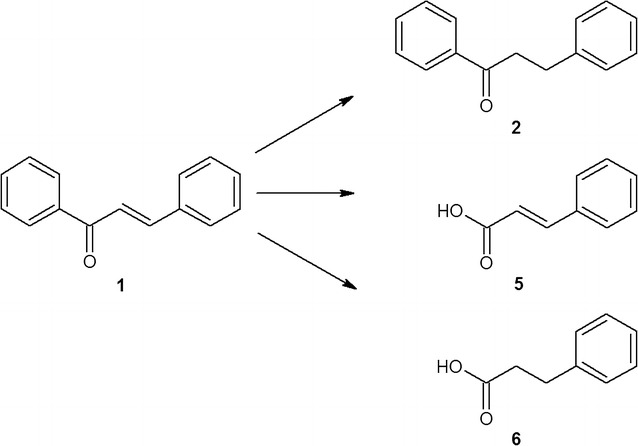
Transformation of chalcone catalysed by *Chroococcus minutus*

Biotransformation of chalcone in the culture of *C. minutus* led to the formation of dihydrochalcone with 70% yield. Moreover, this strain degrades chalcone into two structurally related metabolites, cinnamic and hydrocinnamic acid, with 3 and 10% yields, respectively. The relationship between the amounts of both acids produced suggests that *C. minutus* also catalysed the reduction of the C=C bond in the propenyl chain of the cinnamic acid molecule, which resulted in the appearance of hydrocinnamic acid in the culture (Fig. 3).

The lowest rate of conversion was observed in the case of *S. platensis*, which after 2 weeks of culturing, transformed only 3% of the substrate, producing dihydrochalcone as the only product.

In summary, all the examined strains produced five different products, which were identified based on their relative retention times (Rf values) as well as on their MS spectral data.

The course of biotransformation (in 24 h intervals), i.e., the dynamics of this process, was examined in an axenic culture of *A. klebahnii* as the model of a highly effective biocatalyst. Filamentous, N_2_-fixing *A. klebahnii* is one of the main cyanobacterial strains that forms cyanobacterial blooms, and it is characterized by a relatively rapid increase in biomass [58]. Fortunately, this strain is not reported as being a producer of dangerous cyanotoxins, such as microcystins and cylindrospermopsin [59].

**Fig. 4:**
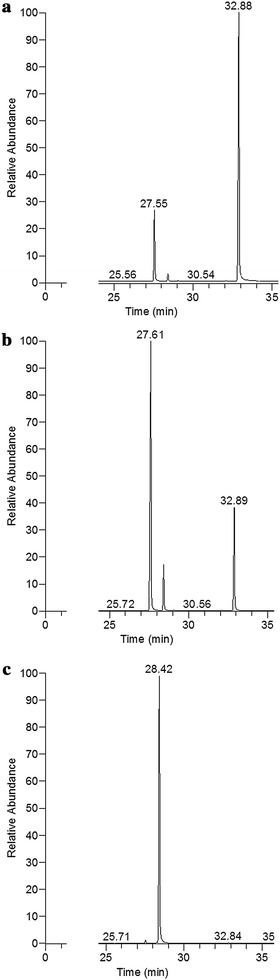
GC–MS profiles of the samples collected on day 0 (**a**), day 1 (**b**), and day 8 (**c**) during the transformation of chalcone into dihydrochalcone by *Aphanizomenon klebahnii*

**Fig. 5:**
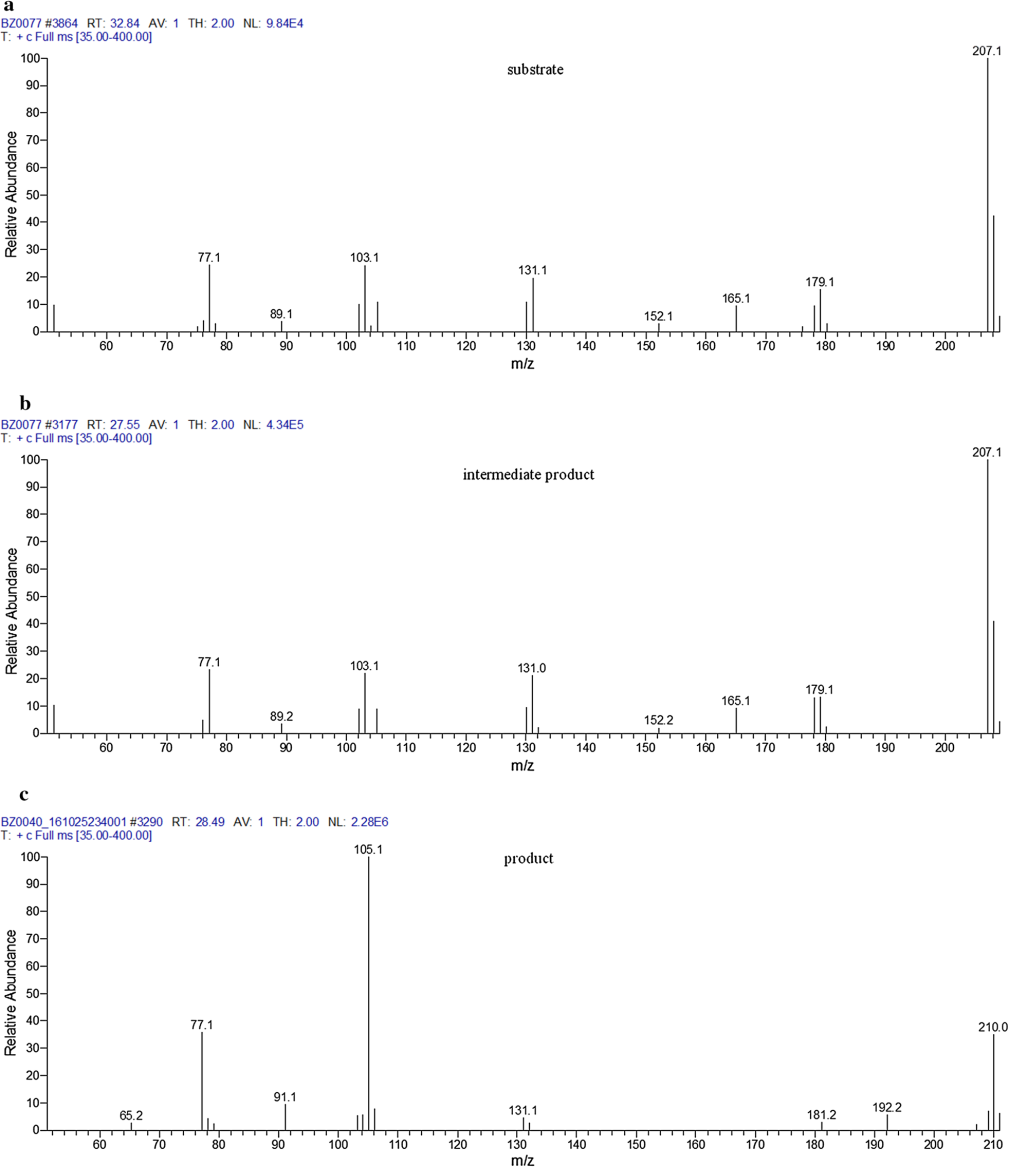
Adequate mass spectra of substrate (**a**), intermediate (**b**) and product (**c**)

This experiment shed the light on the pathway of chalcone conversion as the process proceeded through the intermediate. The results of GC–MS determinations indicated that in both experimental medium and substrate control, during the first 24 h of incubation, the substrate (peak of Rt = 32.8 min) was converted into the intermediate (peak of Rt = 27.6 min). Surprisingly, the m/z fragmentation of this intermediate was identical to that of the substrate (Figs. 4, 5).

**Fig. 6:**
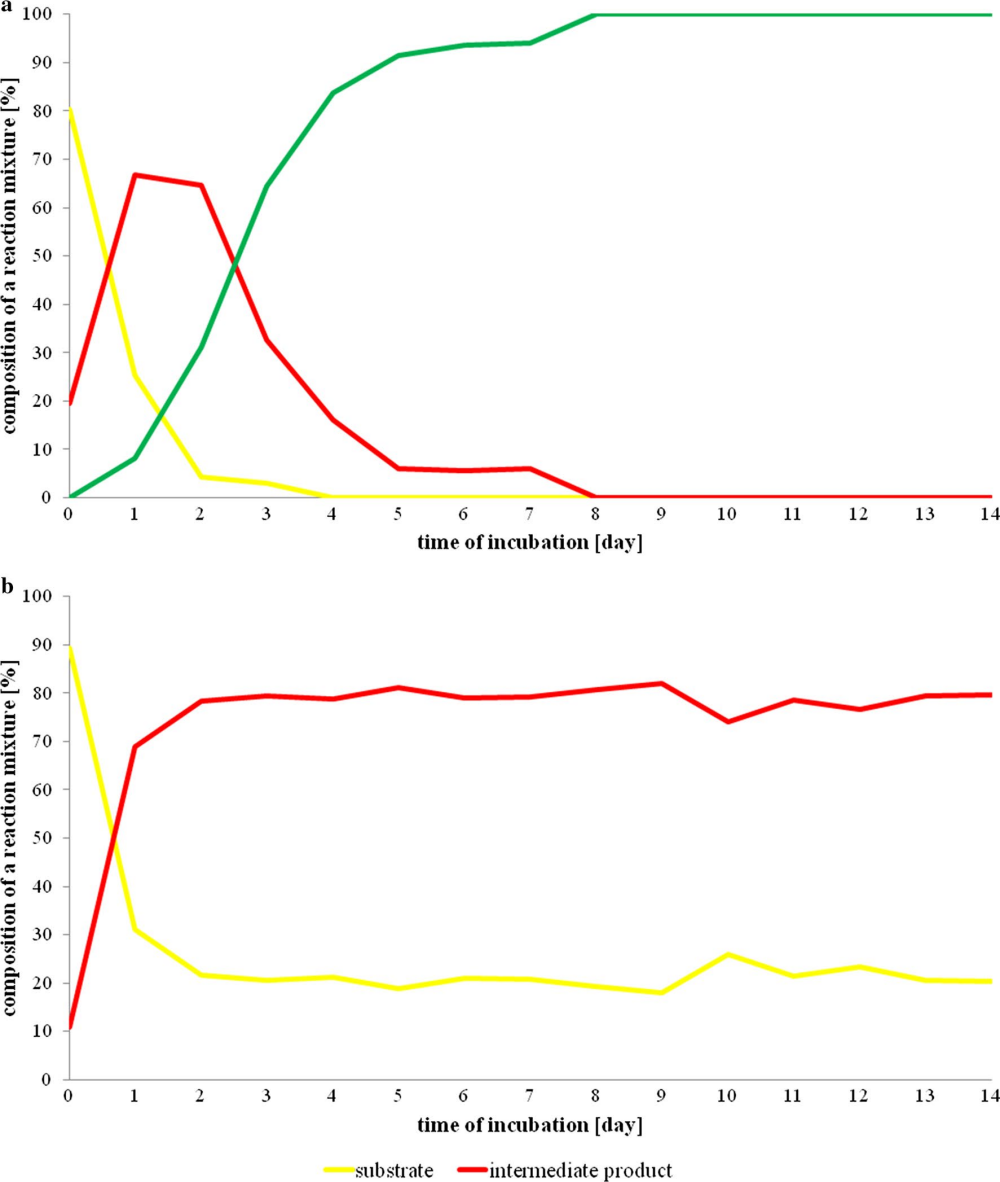
Time-dependent transformations of chalcone in the culture of *Aphanizomenon klebahnii* (**a**) and in substrate control (**b**)

The same intermediate has been detected in substrate control, where it existed in stable equilibrium with *trans*-chalcone, with an 80%:20% ratio after 14 days of incubation (Fig. 6b).

**Fig. 7:**
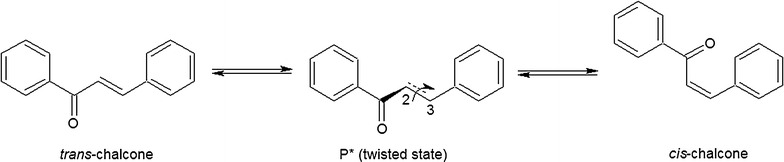
The formation of two distinct, medium-dependent isomeric forms of chalcone

Although chalcone may exist in two isomeric forms, *cis* or *trans*, we presume that it exists as *cis*-chalcone, which might be an intermediate that is then bioreduced by cyanobacteria. It is known that in water solutions irradiated by daylight, *trans*-chalcone is rapidly converted to the excited (twisted) state P*, which forms *cis*-chalcone through a simple isomerization process (Fig. 7) [60].

Such a behaviour of chalcone suggests that, although *trans*-chalcone is the thermodynamically favourable form in polar protic solvents similar to the microbial media, the *cis*-isomer is the dominant form of this compound.

The results of ^1^H NMR measurements of *cis*-chalcone revealed that its spectrum was similar to that of the substrate. Nevertheless, the coupling constant of the H2 and H3 protons of the double bond of the olefin chain was 8 Hz, instead of 15 Hz, which is characteristic of the chalcone of the *trans*-configuration. This result confirmed that *cis*-chalcone is the final intermediate that was transformed by cyanobacteria to dihydrochalcone.

Bearing in mind the dynamics of these transformations, it is worth discussing the sequence of events leading to the complete conversion of *trans*-chalcone. As shown in Fig. 4, the first step was the photochemical transformation of *trans*-chalcone into its *cis*-isomer, which was observed immediately after the addition of the substrate to the medium, even at day 0. The final product of the biocatalytic conversion of *cis*-chalcone, dihydrochalcone (peak of Rt = 28.5 min), was determined the next day together with *cis*-chalcone, whose amount was two times larger than the *trans*-isomer (Fig. 4). From that moment, the biocatalysis proceeded dynamically, alongside a decrease in the concentration of substrate and intermediate up to day 8, when >99% of the substrate and intermediate disappeared. Quantitative data presented in Fig. 6 fully reflect that manner of transformation. Considering the possible enzyme activity, it is worth emphasizing that because of the spatial exposition of the propenyl ketone chain in *cis*-chalcone, this form seems to be more accessible than the analogous group in the *trans*-chalcone molecule. Such a thesis seems to be supported by the data revealing the activity of enoate reductase, which catalyse light-induced, highly selective hydrogenation of C=C bonds [41, 61]. In such circumstances, the catalytic cycle comprises two half-reactions: reduction of flavin mononucleotide by NAD(P)H, followed by flavin oxidation through stereospecific reduction of the C=C bond [62]. Moreover, enoate reductase from anaerobic bacterium *Eubacterium ramulus* can reduce some polyhydroxylated chalcones to adequate dihydrochalcones [63].

The influence of chalcone on the growth of *A. klebahnii* that was monitored daily revealed that the 20 mg/L concentration of chalcone significantly inhibited the growth of examined cyanobacterium. In fact, after 2 weeks, the amount of chlorophyll was approximately 20% of its initial concentration. Nevertheless, such a strong disturbance of microbial growth did not inhibit its biocatalytic activity, which resulted in a >99% conversion of chalcone after 8 days of culturing.

To verify whether such an efficient activity also exists in scaled-up cultures, the same experiments were conducted on a preparative scale and in *A. klebahnii* cultures in a mini-pilot photobioreactor.

### Bioconversion of chalcone by cyanobacteria on a preparative scale

**Fig. 8:**
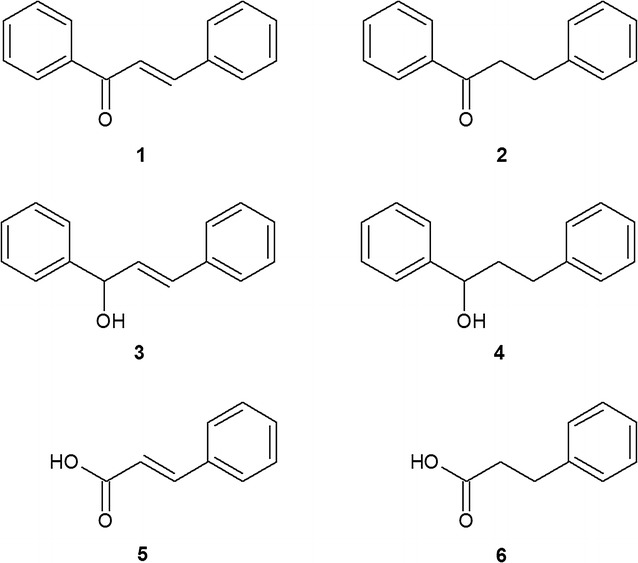
The structures of the substrate—chalcone (**1**) and the products of biotransformation (**2**–**6**) performed by the examined cyanobacteria

Biocatalytic conversion of chalcone on a preparative scale was carried out in 250 mL volumes of cyanobacterial cultures in 1 L Erlenmeyer flasks. The scaled-up bioconversions of chalcone fully confirmed the results obtained on the analytical scale, either with respect to the chemical nature of the products (Fig. 8) or with respect to the transformation dynamics and effectiveness.

Using the preparative scale for the experiments enabled the acquisition of products in amounts that allowed their separation by PTLC and allowed for fully credible confirmation of their structures using a set of spectroscopic methods: MS, IR, and NMR. The effectiveness of biocatalysis in each strain of cyanobacterium was established based on quantitative determination using mass spectrometry coupled with gas chromatography (Table 1). Table 2 presents the GC–MS data for compounds **1**–**6**.

**Table 1:** The yields of biocatalytic transformation of chalcone by cyanobacteria examined in experiments carried out on a preparative scale

Strain of cyanobacteria	Product	Yield (%)
*Spirulina platensis*	**2**	3.1 ± 0.2
*Anabaena laxa*	**2**	>99.0
*Anabaena* sp.	**2**	87.6 ± 1.2
	**3**	6.7 ± 1.5
	**4**	5.7 ± 0.2
*Aphanizomenon klebahnii*	**2**	>99.0
*Nodularia moravica*	**2**	>99.0
*Chroococcus minutus*	**2**	69.9 ± 1.9
	**5**	2.9 ± 0.4
	**6**	9.8 ± 2.5
*Merismopedia glauca*	**2**	91.8 ± 5.0
*Synechocystis aquatilis*	**2**	>99.0

The yields are the average values from at least four repetitions

**Table 2:** GC–MS data for compounds **1**–**6**

No.	Compound	RT (min)	Fragment ions
**1**	Chalcone	32.80	208.1 [M]^+^, 207.1, 179.2, 165.1, 152.2, 131.1, 103.1, 89.2, 77.1
**2**	Dihydrochalcone	28.40	210.1 [M]^+^, 106.0, 105.0, 91.2, 77.1, 51.0
**3**	1,3-Diphenyl-2-propen-1-ol	30.20	208.1 [M]^+^, 121.1, 105.1, 91.1, 77.1, 51.1
**4**	1,3-Diphenylpropan-1-ol	28.20	208.1 [M]^+^, 107.1, 92.1, 79.0, 65.1, 51.0
**5**	Cinnamic acid	13.60	148.1, 147.1 [M]^+^, 131.1, 119.1, 103.1, 91.1, 77.1, 51.2
**6**	Hydrocinnamic acid	10.60	150.0 [M]^+^, 131.1, 104.1, 91.2, 78.1, 65.1, 51.1

Although halophilic species of cyanobacteria grow faster and more efficiently than freshwater species, but are less efficient biocatalysts in case of chalcone transformation. The most probable reason is related to the transmembrane transport, resulted from the specific structure of the cell wall of halophilic cyanobacteria as *S. platensis* is [64]. For this reason, transmembrane transport in the cells of halophiles strains is better controlled, what means that the transportation of uncertain substances (as chalcone and/or dihydrochalcone are in this case) is carried out to the smaller extent. Therefore the differences in a cell wall construction and physiological features of halophilic and freshwater cyanobacteria, may be the reasons for their various biocatalytic abilities. Additionally, consistent physiological differences between freshwater and marine taxa correspond to clear genetic divergence and possible differences in cell division. The main distinction is intolerance of moderate to high salinities in freshwater taxa, while marine taxa have no problem growing in freshwater [65]. Even if mentioned *S. platensis* is not the most effective biocatalyst in formation of dihydrochalcone, for sure is safe for consumers and its activity provided the added value to its use, simply as it is—without any modifications. We think that this strain is the example that might fulfill together the requirements of pharmaceutical and food industry as commonly accepted nutraceutic.

### Bioconversion of chalcone by *Aphanizomenon klebahnii* carried out in a mini-pilot scale photobioreactor

For 2 L volume batch cultures, the proposed regimen of intensity and the colour of light, pH, temperature, vigour of mixing and aeration of medium, allowed for biocatalysis conducted by *A. klebahnii* in Z8 medium to be shortened from 14 to 4 days. What should be emphasized is that the yield of this scaled-up transformation was >99%, which is the same as the conversion yields at the analytical and preparative scales. Fourteen-day transformation of chalcone (**1**) (40 mg) in 2 L volume of the culture of *A. klebahnii* yielded 30 mg of compound **2**, with at least over 75% absolute productivity. It is worth to emphasize that this system has not been optimized yet.

**Fig. 9:**
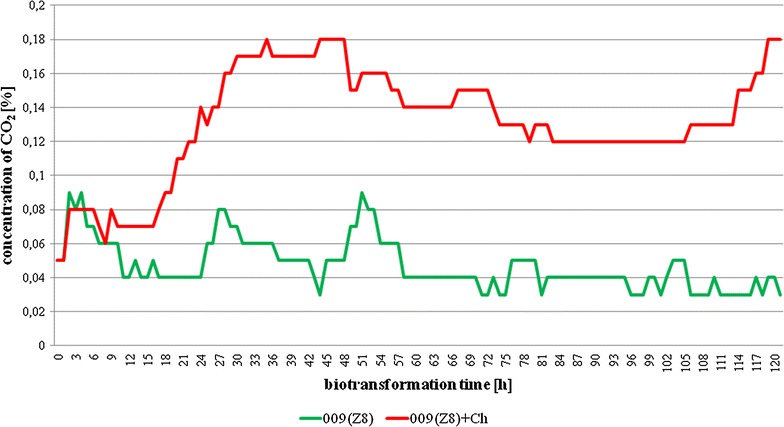
The changes of CO_2_ content in the headspace of the photobioreactor (b) as a function of time in control and experimental cultures of *Aphanizomenon klebahnii*

Interestingly, such a high effectiveness of conversion was accompanied by relatively low photosynthetic activity for the cyanobacterium, confirmed by an approximately constant pH (7.0 ± 0.2) throughout the culture period. However, in the case of the control cells, the pH fluctuated since it was influenced by exponentially increasing photosynthetic CO_2_ fixation during the dark periods and cyanobacterial respiration (responsible for CO_2_ releasing) when the light was operated (Fig. 9). Moreover, the increase in respiration rate, indicated by higher CO_2_ concentration over the surface of the medium, was more pronounced in the experimental culture, which suggests the higher activity of ongoing catabolic processes.

In the closed system of the batch photobioreactor culture, the changes in pH also resulted from a steady decrease in the amount of nutrients, from net fluxes of H^+^ across the plasma membranes, and from the influx of weak electrolytes such as CO_2_/HCO_3_^−^ and NH_4_^+^/NH_3_ [56]. The inorganic carbon equilibrium is easily unbalanced because uncatalysed hydration–dehydration reactions are very slow compared to biological interactions in carbon assimilation and/or releasing, in which case cyanobacteria may catalyse by carbonic anhydrase. Many strains of blue-green algae can actively transport inorganic carbon from the growth medium into cells when passive CO_2_ diffusion is not adequate to support photosynthetic demands. Moreover, in a closed system, a dense blue-green algal culture can profoundly influence the pH of the medium since cyanobacteria has only a limited pH range within which growth is possible and usually has a defined pH optimum [66].

Microbial synthesis of flavonoids is well documented in the literature; however, there are no reports on the chemomicrobial synthesis of dihydrochalcones using cyanobacterial strains. It seems logical that organisms producing flavonoids possess a suitable “enzymatic apparatus” to transform such natural compounds, with specific enzyme behaviour advantages, such as chemo-, regio-, and stereoselectivity. It is possible that the cells can adjust such a system to a specific substance that appears in their environment through the formation of enzymes enabling the metabolism of this compound and its inclusion in the chain of internal changes. When phototrophic microorganisms such as cyanobacteria are exposed to the influence of xenobiotic compounds, they may develop the capacity for metabolic adaptations. In this respect, cyanobacteria can perform an incomplete degradation of the substrate, which may reduce the probability of by-product formation or further product degradation [67].

The metabolic capabilities of cyanobacteria create a rational premise to select the conditions of biocatalytic transformations of chalcones that enable targeted biotransformations based on the structural variability of these compounds. One of these processes may be the formation of dihydrochalcone, a valuable natural sweetener. Starting material supplying the chalcones for bioconversion to the desired products may be a by-products from other branches of industry, e.g. hop plant *Humulus lupulus*. The residue after hop supercritical carbon dioxide extraction is a rich source of technologically useful substances, primarily chalcones, as described by Anioł [68]. Other well-known plants rich in chalcones are cherry tomatoes [69], kava–kava (*Piper methysticum*) [70], *Boesen*-*bergia rotunda* [71], and *Lophira alata* [72].

### Chromatographic and spectroscopic data about biotransformation products

#### Dihydrochalcone/1,3-diphenylpropan-1-one (**2**)

Chalcone (**1**) was used as a substrate. The crude product was purified by preparative thin layer chromatography (hexane:acetone 3/1, v/v) to obtain white crystals, mp. 71–73 °C; ^1^H NMR (400 MHz) (DMSO-d^6^) δ (ppm): 7.99 (d, 2H, J = 7.6 Hz), 7.60 (t, 1H, J = 7.6 Hz), 7.50 (m, 2H, J = 8 Hz), 7.28 (d, 4H, J = 7.6 Hz), 7.17 (d, 1H, J = 7.2 Hz), 3.37 (t, 2H, *J* = 8,0 Hz), 2.94 (t, 2H, J = 7.6 Hz); ^13^C NMR (100 MHz) (DMSO-d^6^) δ (ppm): 199.1, 141.2, 136.5, 133.1, 128.6, 128.3, 127.9, 125.8, 39.5, 29.4; IR (KBr) νmax/cm^−1^ 3061, 2927, 2341, 1957, 1824, 1681, 1595, 1494, 1448, 1208, 1075, 973, 928, 743, 701; GC–MS (EI) *m/z* (%) = 210.1 (33) [M]^+^, 106.0 (12), 105.0 (100), 91.2 (10), 77.1 (39), 51.0 (10); the retention times: 1–32.80, 2 –28.40 min.

#### 2-Propenyl, 1-hydroxy-1,3-diphenyl-/1,3-diphenyl-2-propen-1-ol (**3**)

Transformation of chalcone (**1**) in the *Anabaena* sp. culture yielded compound 3 (white solid); mp. 55–57 °C; ^1^H NMR (400 MHz) (CDCl_3_) δ (ppm): 7.26–7.40 (m, 10H), 6.66 (dd, 1H), 6.40 (m, 1H), 5.14 (m, 1H), 1.61 (s, 1H); ^13^C NMR (100 MHz) (CDCl_3_) δ (ppm): 142.8, 136.5, 131.5, 130.6, 128.6, 128.5, 127.8, 127.7, 126.6, 126.3, 75.1; IR (KBr) νmax/cm^−1^ 3345, 3027, 1599, 1493, 1450, 1010, 966, 746, 696; GC–MS (EI) *m/z* (%) = 208.1 (42) [M]^+^, 121.1 (21), 105.1 (100), 91.1 (57), 77.1 (61), 51.1 (15); the retention time: 3–30.20 min.

#### 1,3-Diphenylpropan-1-ol (**4**)

Transformation of chalcone (**1**) in the culture *Anabaena* sp. yielded compound 4 (pale yellow oil); mp. 52–54 °C; ^1^H NMR (400 MHz) (CDCl_3_) δ (ppm): 7.37–7.21 (10H, m), 4.70 (1H, m), 2.83–2.61 (2H, m), 2.22–1.97 (2H, m); ^13^C NMR (100 MHz) (CDCl_3_) δ (ppm): 144.6, 141.9, 128.6, 128.5, 128.4, 127.7, 126.0, 125.9, 73.9, 40.5, 32.1; IR (KBr) νmax/cm^−1^ 3395, 3027, 2940, 1603, 1495, 1454, 1058, 747, 699; GC–MS (EI) *m/z* (%) = 208.1 (4) [M]^+^, 107.1 (80), 92.1 (61), 79.0 (100), 65.1 (13), 51.0 (12); the retention time: 4–28.20 min.

#### 2-Propenoic acid, 3-phenyl/cinnamic acid (**5**)

Transformation of chalcone (**1**) in the *C. minutus* culture yielded compound **5** (white crystals); mp. 132–133 °C; ^1^H NMR (400 MHz) (DMSO-d^6^) δ (ppm): 12.41 (s, 1H), 7.69–7.67 (m, 2H), 7.60 (d, 1H, J = 8 Hz), 7.42–7.40 (m, 3H), 6.54 (d, 1H, J = 8 Hz); ^13^C NMR (100 MHz) (DMSO-d^6^) δ (ppm): 168.4, 144.7, 135.1, 133.1, 129.7, 129.1, 120.1.; IR (KBr) νmax/cm^−1^ 3230–2485, 1692, 1625, 1450, 1288, 982, 769, 712; GC–MS (EI) *m/z* (%) = 148.1 (58), 147.1 (100) [M]^+^, 131.1 (15), 119.1 (4), 103.1 (45), 91.1 (18), 77.1 (22), 51.2 (14); the retention times: 5–13.60 min.

#### Benzenepropanoic acid/hydrocinnamic acid (**6**)

Transformation of chalcone (**1**) in the *C. minutus* culture yielded compound **6** (colourless to faintly yellow); mp. 45–48 °C; ^1^H NMR (400 MHz) (CDCl_3_) δ (ppm): 7.21–7.33 (m, 5H), 2.97 (t, 2H, J = 7.8 Hz), 2.70 (t, 2H, J = 7.8 Hz); ^13^C NMR (25 MHz) (CDCl_3_) δ (ppm): 179.6, 140.2, 128.6, 128.3, 126.4, 35.7, 30.6; IR (KBr) νmax/cm^−1^ 3060–2633, 1699, 1603, 1409, 1304, 1221, 933, 703; GC–MS (EI) *m/z* (%) = 150.0 (45) [M]^+^, 131.1 (4), 104.1 (100), 91.2 (81), 78.1 (18), 65.1 (14), 51.1 (10); the retention times: 5–10.60 min.

## Conclusions

Cyanobacterial cultures are ready to be used for highly efficient chemoselective conjugate reduction of α,β-unsaturated carbonyl compounds occurring in aqueous media in mild conditions. This is the first report related to the use of whole cyanobacterial cells for the bio-reduction of 1,3-diaryl-2-propen-1-one, producing the corresponding saturated ketone; 1,3-diaryl-2-propan-1-one. After optimization of this process, the complete conversion of substrate (>99%) was obtained. The use of blue-green algae as biocatalysts in the biotransformation of chalcones seems to be an important biotechnological strategy to obtain dihydrochalcone with high chemoselectivity, using low-cost reagents. Moreover, the scaling-up of this process to preparative and mini-pilot scales fully corroborates the results obtained with the analytical scale. Moreover, >99% effectiveness of bioconversion on analytical, preparative, and mini-pilot scales creates the possibility of rescaling this process to a semi-technical or technical scale. Therefore, we propose a relatively simple way of obtaining dihydrochalcone. Due to its sweet taste and growing interest in its use, this method may find an application in the food industry that is still eager for the production of new, natural sweeteners.
